# Caregivers’ experiences of being asked about adverse childhood experiences and receiving support from an integrated health and social care hub: a qualitative study

**DOI:** 10.1136/bmjopen-2024-086710

**Published:** 2025-02-05

**Authors:** Ashraful Kabir, Sarah Loveday, Harriet Hiscock, Rebecca Bosward, Wilhelmina Ebbett, Hueiming Liu, Natalie White, Lingling Chen

**Affiliations:** 1 Health Services, Murdoch Children's Research Institute, Parkville, Victoria, Australia; 2 The Royal Children’s Hospital, Parkville, Victoria, Australia; 3 Sydney Local Health District, Sydney, New South Wales, Australia; 4 The George Institute for Global Health, Camperdown, Sydney, Australia

**Keywords:** Health Services, MENTAL HEALTH, Community child health, Psychosocial Intervention, QUALITATIVE RESEARCH

## Abstract

**Abstract:**

**Objectives:**

Adverse childhood experiences (ACEs) are significant contributors to the burden of disease and remain a serious concern for the health and wellbeing of children in Australia. To address ACEs, we co-designed and implemented two integrated health and social care hubs (Child and Family Hubs [CFHs]). This study explores the experiences of caregivers who received care from the CFHs, including the way they were asked about ACEs and the services offered to address identified ACEs.

**Design:**

A qualitative study design was used. Using a semistructured interview guide, 29 in-depth interviews were conducted with caregivers of children who were experiencing a range of adversities, including maltreatment and household dysfunction, child neglect, parent mental illness, domestic violence, family conflict, community dysfunction, discrimination, poverty or financial hardship. A thematic analysis approach was used to analyse textual data. Triangulation of investigators and sources of data improved validation of the findings. NVivo (V.12) was used to organise, index and retrieve data.

**Settings:**

This study was conducted in two Child and Family Hubs (CFHs) in Australia—IPC Health, Wyndham Vale, Melbourne, and Marrickville Health Centre, Sydney, between May and October 2023.

**Participants:**

Participants (n=29) were the caregivers of children living with adverse childhood experiences (ACEs).

**Results:**

Four themes were identified which reflected the caregivers’ experiences of being asked about adversities and how they linked to the support and services both in the CFHs and outside the CFH. These themes were as follows: (i) trusting relationships are fundamental; (ii) expectations play a role in talking about adversities; (iii) barriers to open discussion of adversities and (iv) barriers to accessing services.

**Conclusions:**

Consultations between caregivers and hub practitioners can effectively identify and address ACEs despite certain barriers. Establishing a trusting relationship where caregivers feel heard and supported is vital, highlighting the hub model’s potential impact in Australia and similar contexts. Enhancing consultation duration, and service availability and accessibility may further improve caregivers’ experiences in identifying and addressing adversity.

**STRENGTHS AND LIMITATIONS OF THIS STUDY**

- The use of semistructured interviews allowed for an in-depth exploration of participants’ experiences and views.
- Data were collected from multiple sites, which enhanced the diversity of perspectives and contributed to a comprehensive understanding of the phenomenon under study.
- A thematic analysis approach was employed for data analysis, providing a detailed interpretation of participants’ experiences, while triangulation of data sources and investigators increased the trustworthiness of the analysis.
- As a qualitative study, the findings may be limited in terms of transferability due to the contextual characteristics of the study settings.

## Background

 Adverse childhood experiences (ACEs) are significant contributors to the burden of disease and pose severe threats to the health and well-being of children and adolescents globally.^1 2^ Traditionally, ACEs are defined as stressful circumstances or events that children may face while growing up, such as maltreatment (eg, physical, emotional or sexual abuse) and household dysfunction (eg, parental mental illness and family substance abuse). More recently, the definition of ACEs has been broadened to family adversity—an umbrella term that includes events outside the child’s home, that is, community dysfunction (eg, witnessing physical violence and discrimination), bullying and socioeconomic deprivation.^3 4^ A growing body of research has demonstrated that individuals exposed to ACEs and family adversity are more likely to have poorer physical and psychosocial outcomes than those not exposed.^5^,^8^

In Australia, ACEs remain a significant public health problem,^9 10^ despite an increase in ACE-related services.^10^ Around 64% of children attending a community paediatric clinic in South-Western Sydney reported having one or more ACEs,^11^ while a national study reported that one in every five children had experienced three or more ACEs,^12^ and more than half of the children studied experienced at least two adversities by the age of 10–11 years.^13^ Furthermore, ACEs are unevenly distributed among children from ethnic minority and indigenous backgrounds with low socioeconomic positions being four to eight times more likely to be exposed to two or more ACEs than those from Anglo-European or wealthier backgrounds.^13^

A recent review indicates that integrated models of care that bring health and social care practitioners together can improve mental health outcomes for children who are experiencing adversity.^14^ In recognition of the importance of integrated models of care, Child and Family Hubs (CFHs)—designed to integrate health and social care as key responses to adversity—have been proliferating in Australia.^15^,^17^ A key component of this care model is identifying service users who have experienced ACEs and addressing their needs to improve their conditions, such as material conditions (eg, access to housing and welfare payment), immigration-related legal and financial issues, and physical and mental health of the caregiver and child.^18^ In this process, effective communication—beyond the routine inquiry about ACEs—between service users (eg, caregivers) and service providers (eg, general practitioners, paediatricians, nurses, well-being coordinators, lawyers and financial counsellors) is essential. Effective communication refers to the process of sharing information between the service users (eg, patients) and the service providers, which also encompasses interactions with the patient’s family and caregivers. It is recognised that routine inquiries about ACEs are essential for effective communication within integrated care models. By consistently asking about these experiences, service providers convey that caregivers’ experiences are valued, fostering trust and rapport. This approach encourages caregivers to actively participate and collaborate in the care process, making effective communication and routine questioning closely linked concepts. Such communication can enhance service users’ engagement in the care-seeking process,^19 20^ foster collaborative relationships and shared decision-making between service users and providers,^21 22^ aid in identifying health issues, develop treatment pathways and improve adherence to care outcomes.^23 24^

Previous studies have explored healthcare providers’ perspectives on inquiring about ACE-related issues in healthcare settings.^25 26^ These studies indicate that healthcare providers are reluctant to ask service users about ACEs due to a range of issues including a perceived lack of time, and lack of confidence, training and comfort in directly asking about and responding to childhood adversity.^27 28^ Practitioners have a limited understanding of the impact of personal biases on their understanding and recognition of adversity.^22^ Further issues include insufficient resources required to implement ACE-specific interventions,^29 30^ and the use of ineffective tools and approaches to screening in primary-level care settings.^31^ In contrast, limited research has been conducted from the perspectives of service users in their experiences of being asked about family adversities during care-seeking. It has been indicated that service users are likely reluctant to voluntarily disclose adversities to service providers due to discomfort, shame, upset and distress.^32^ However, previous studies focused on consultations asking about adversity during care-seeking are quantitative and/or conducted in clinical settings.^33^ Therefore, there are information gaps in how caregivers are being asked about ACEs at health and social care hubs, how feasible and acceptable this practice is to the caregivers, and how they experience receiving support from CFHs. Thus, this study explores caregivers’ experiences of being asked about ACEs and the services offered, as well as the additional services required to address ACEs in two CFHs in Melbourne and Sydney, Australia. The findings may be useful to better implement the current integrated health and social care hub model of care and inform the future operation of CFHs in Australia and elsewhere.

## Materials and methods

### Study and setting

This study was conducted as part of evaluating co-designed CFHs to better detect and respond to ACEs.^33^ The CFHs were based at IPC Health, Wyndham Vale, and Marrickville Health Centre, Sydney. The data from both sites were combined as a single dataset, focusing on identifying facilitators and barriers to openly discussing ACEs and accessing relevant support. The Wyndham Vale CFH’s workforce includes paediatricians, general practitioners, nurses, a family violence worker, a speech pathologist, a dietician, a well-being coordinator, a financial counsellor and lawyers. Wyndham Vale is a rapidly growing suburb of greater Melbourne and has a large proportion of the population who is culturally diverse and from immigrant backgrounds.^34^ This diversity is often associated with higher levels of economic and social vulnerability, such as insufficient employment opportunities, lack of social cohesion, higher levels of social isolation and reduced community connectivity. Nearly one-quarter of children living in Wyndham Vale and commencing school are estimated to be more vulnerable in at least one developmental domain compared with less than one in ﬁve children across Victoria.^35^ Mental health problems (including depression or anxiety) are recorded as the most common long-term health condition experienced by 8.5% of the population.^36^

Marrickville Health Centre offers an array of services, and the CFH workforce includes community paediatricians, speech pathologists, an occupational therapist, child psychologists, dieticians, social workers, as well as a service navigator and lawyers. Marrickville is a suburb in the Inner-West of Sydney which is culturally diverse and made up of populations from a wide range of countries, including Vietnam, Greece, England, New Zealand and China. Aboriginal and Torres Strait Islander Peoples make up 1.6% of the population. Approximately 11.3% of people in Marrickville reported experiencing a long-term mental health condition, including depression and anxiety in the 2021 census.^37^

**Figure 1:**
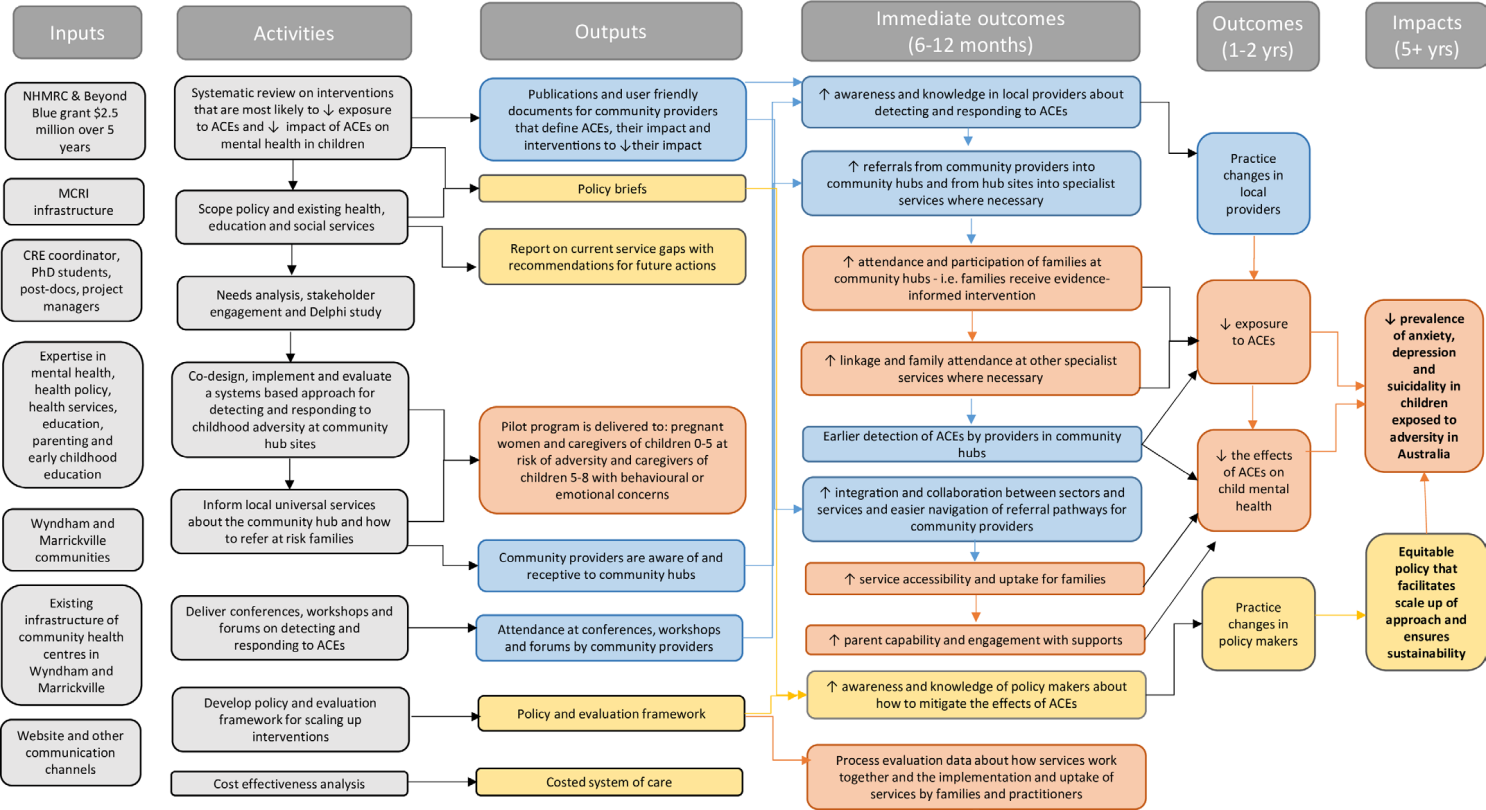
Child and Family Hub (CFH) logic model of care. ACEs, adverse childhood experiences; CRE, Centre of Research Excellence; MCRI, Murdoch Children’s Research Institute; NHMRC, National Health and Medical Research Council.

The CFH initiative aimed to improve child mental health by identifying and addressing adversities faced by families with children aged 0–8 years. This broader project involved co-designing, implementing and evaluating CFH models at two sites, focusing on their impact on caregivers’ ability to identify adversities and make service referrals as seen in figure 1.^18^ The initiative raised local providers’ awareness of ACEs and enhanced referral pathways to community hubs and other services. Key activities included training community providers to recognise and respond to ACEs and facilitating referrals within and beyond the CFH. Early detection was intended to increase family participation in CFH, promote collaboration across sectors and enhance service accessibility. Additionally, the programme promoted parent engagement, raised awareness among policymakers about ACE mitigation and encouraged equitable policies for sustainable scaling. The model also evaluated implementation and outcomes to guide policy changes for sustainability and scalability.^18^

### Study participants and sampling strategy

Interviews were conducted between May and October 2023 with caregivers living in Wyndham Vale and Marrickville. Participants were selected using the following inclusion criteria: received care from the CFHs within the past 12 months, had children aged 0–8 years who attended the Hub, experienced one or more adversity (as reported in the baseline survey), availability and voluntary participation. We employed purposive sampling to select the participants. In this process, the maximum variation in terms of the participant’s age, gender, occupation and adversities faced was ensured. In the initial step, we approached potential participants who had participated in our baseline Hub survey and indicated in the survey that they were happy to be contacted for an interview. This survey was conducted as part of formative research of the hub.^38^ The baseline survey recruited a total of 349 caregivers (Victoria: n=234; NSW: n=115). Across both sites, 88% reported experiencing one or more family adversities across various domains. Over half reported experiencing adversity outside the home, and 80% reported at least one form of adversity inside the home. Furthermore, in Victoria and NSW, almost 40% and 30%, respectively, experienced five or more adversities of any type.^22 38^

In Wyndham Vale, a researcher (LC) contacted these caregivers, explained the study’s objectives and purpose and invited them to participate in the interview. In Marrickville, a researcher (RB) contacted participants using the information they provided as part of the baseline survey and invited them to participate in an interview. Based on the literature and research team experience in similar contexts and issues, we planned to conduct 36–40 interviews to reach data saturation, as described in our study protocol.^18^ We achieved data saturation by the 29^th^ interview, as no new information, dimensions or concepts emerged.^39^

### Data collection procedure

A team of six researchers (LC, SL and AK in Wyndham Vale, and RB, WE and HL in Marrickville) implemented the data collection and analysis. LC is an experienced qualitative researcher in psychology. SL is a paediatrician with extensive experience in qualitative research and AK is a public health practitioner with extensive experience in qualitative research. RB is a research coordinator with experience in qualitative research in public health and healthcare. WE is a clinical research nurse with experience in qualitative research. HL is a Senior Research Fellow with extensive experience in qualitative research. A semistructured interview guide was used to collect data (online supplemental file 1). It was piloted with lived experienced representatives outside the hub catchment areas in advance, and necessary adjustments were made. Based on the advice from lived experience researchers during CFH co-design, the term ‘life challenges’ was used throughout the interviews instead of ‘adversity’. The interviews explored caregivers’ experiences, views and opinions regarding being asked about ‘life challenges’ by CFH practitioners and the services offered, as well as the additional services required to address challenges. Interviews were conducted in English, either in-person at the CFHs, online or by telephone depending on the participant’s preference. Rapport was established before each interview, and formal electronic written consent was obtained prior to audiorecording of the interview. In Marrickville, verbal consent was obtained from participants at the time of interview, audio recorded and transcribed into an official consent form. LC and AK conducted all 20 interviews at Wyndham Vale, which averaged 54 min (range 25–113 min). RB and WE conducted 12 interviews at Marrickville. Three participants were excluded during the interview process who had not visited Marrickville in the past 12 months, resulting in nine interviews, averaging 23.4 min (range 15–39). Although the average duration of interviews varied between sites (54 vs 23 min), we did not observe that this substantially impacted the themes that emerged. However, we acknowledge this difference as a minor limitation that may have influenced the depth of insights captured.

### Data analysis

A thematic analysis approach was employed to analyse and present the data.^40^ NVivo (V.12) was used to organise, index and retrieve data. Guided by the research objectives, three researchers (LC, SL and AK) developed an initial coding framework by independently reading a subset of transcripts. To increase the trustworthiness of the coding process, the individual coders then cross-checked and reflected each other’s coded transcripts with each transcript coded by two coders independently. Formal discussions among the coders were held to resolve disagreements and thus reach a consensus. The thematic analysis approach produced a rich description of codes and themes. The research team followed a stepwise procedure to perform the thematic analysis: (a) familiarising themselves with the textual data through repeated reading; (b) generating initial codes and collating the textual data in designated codes; (c) reviewing and sorting all coded data and formatting a thematic matrix; (d) collapsing the codes and labelling the final themes and subthemes and (e) reporting findings under the themes. The Consolidated Criteria for Reporting Qualitative Studies, a 32-item checklist, was followed to report findings.^41^

### Patient and public involvement

The larger study had extensive participation through co-design and lived experience involvement which influenced the design of this study and the CFH. This included CFH consumer involvement to ensure the term ‘life challenges’ was used throughout our evaluation and not ‘ACEs’ or ‘adversity’.

## Results

### Participant characteristics

Table 1 summarises participant characteristics. In Wyndham Vale, the mean age of participants was 36 years (SD=7.77; range=25–59) and two-thirds were female. Half of the participants were born in Australia, and over half (11/20) reported English as their primary language. Participants were well-educated with most participants completing high school or further higher education. Almost half of the participants (9/20) reported having two children, and over half (12/20) lived with their partners and children. The mean age of participants in Marrickville was 37 years (SD=5.50; range=30–46), and all participants were female. Most were born in Australia and reported English as their primary language (8/9). Participants commonly had a bachelor’s degree (7/9), with all participants having a twelfth-grade education or higher. Most participants had one child (7/9) and resided with their partners and children (7/9). The difference in participant demographic characteristics between the two CFHs was not observed to significantly impact the themes that emerged or the overall analysis.

**Table 1:** Characteristics of interview participants (N=29)

Characteristics	Study sites		Total (N=29)
	Wyndham Vale, N=20	Marrickville**, **N=9	
	n (%)	n (%)	
**Age, mean (SD, range**)	36 (7.77, 25–59)	37 (5.5, 30–46)	36.4 (7.2, 25–59)
Not disclosed	0	1 (11)	1 (3)
**Gender**			
Female	15 (75)	9 (100)	24 (83)
Male	5 (25)	0	5 (17)
**Country of birth**			
Australia	10 (50)	6 (67)	16 (55)
India	4 (20)	0	4 (14)
Pakistan	1 (5)	0	1 (3)
Ethiopia	1 (5)	0	1 (3)
China	1 (5)	1 (11)	2 (7)
Mongolia	1 (5)	0	1 (3)
Togo	1 (5)	0	1 (3)
Sierra Leone	1 (5)	0	1 (3)
Italy	0	1 (11)	1 (3)
New Zealand	0	1 (11)	1 (3)
**Primary language spoken**			
English	11 (55)	8 (89)	19 (66)
Other	9 (45)	1 (11)	10 (34)
**Aboriginal and Torres Islanders**			
Yes	1 (5)	0	1 (3)
No	19 (95)	9 (100)	28 (97)
**Highest level of schooling**			
Year 10	1 (5)	0	1 (3)
Year 12 (final school year)	6 (30)	2 (22)	8 (28)
Trade or other certificate-level qualification	3 (15)	0	3 (10)
Bachelor’s degree	5 (25)	7 (78)	12 (41)
Postgraduate qualification	5 (25)	0	5 (17)
**Number of children in the household**			
1	4 (20)	7 (78)	11 (38)
2	9 (45)	1 (11)	10 (34)
3	6 (30)	1 (11)	7 (24)
4	1 (5)	0	1 (3)
**Co-residents (living with whom**)			
Partner and children	12 (60)	6 (67)	18 (62)
Children only	7 (35)	1 (11)	8 (28)
Extended family and children	1 (5)	1 (11)	2 (7)
**Adversities reported[Table-fn T1_FN1]**			
Range of adversities	3–10	4–9	3–10
Total number of adversities	26	19	45
**Average duration of interviews (min**)	54 (25–113)	23 (15–39)	39 (15–113)

* Adversities included challenges outside of the families, family physical health, parental mental health challenges, parenting challenges, child neglect, child abuse, family relationship challenges, family violence, alcohol and drug challenges, and broader societal needs.

### Caregivers’ experiences with enquiries about ACEs and accessing referred support

Four themes were developed, reflecting caregivers’ experiences when discussing adversities and accessing referred support and services. First, a trusting relationship between caregivers and practitioners provided a foundation for open discussions about family adversities. Within this relational framework, caregivers’ expectations of the consultation influenced whether, what and how much they were willing to share. While some caregivers were open to discussing their adversities, barriers such as reluctance to speak openly in front of their children and limited consultation time impeded full disclosure. Additional challenges in accessing referred support included caregivers’ limited resources and long wait times for medical and mental health services.

#### Trusting relationships are fundamental

Trusting relationships and understanding between caregivers and practitioners emerged as the fundamental basis for creating a safe interpersonal space for caregivers to openly discuss family adversities and increase their likelihood of accepting referrals. Caregivers in both sites stated,

I had a really good relationship with her. I trusted her, and we spoke a lot… she always gave me really good feedback (a participant from Wyndham Vale), and We trust her a lot. It makes us trust her advice quite a lot as well (a participant from Marrickville).

Trusting relationships were largely fostered through a relational approach undertaken by practitioners, such as attentive and nonjudgemental listening, as one participant from Wyndham Vale reflected:

I have been seeing Dr. X since 2019. It’s almost four and a half years since we are seeing her. And the way she listens and personally, that person, you get to the point where you feel like that person is listening to you and understanding you instead of just, some people just make you feel judged. So, because with Dr. X, I feel very comfortable and not being judged on the way I was thinking about my family and my situation, that’s why I had trust in her. And I’m happy to trust her in anything.’

Not listening attentively could undermine caregiver-practitioner relationships and result in caregivers’ reluctance of sharing life challenges, as illustrated by a participant from Marrickville:

As I started recognising that she wasn’t listening to what I was saying, she asked some pretty insensitive questions that made it clear that she missed the point of what I was saying. And I think as you start feeling like someone is misunderstanding you and thinking, like, getting the wrong idea about what’s happening in your personal life, I think I got a bit more stressed and agitated and upset.’

Practitioner characteristics, such as gender, language and cultural background, also influenced the level of trust in caregiver-practitioner relationships. Caregivers felt that shared characteristics fostered a sense of understanding and relatability. For example, one participant from Wyndham Vale explained:

It’s easy to understand if a person belongs to your own culture, so it’s easy to get into their shoes. Of course, everything in every culture is definitely, and of course it is different, but it’s easy to talk to her because it’s mainly because of the language; there’s no language barrier.’

Framing ACE inquiries within a whole-person approach—considering caregivers and their family’s physical, mental and broader social contexts—encouraged disclosure of family adversities. Caregivers felt that this approach reflected practitioners’ genuine concern and understanding of their challenges beyond health issues, including family dynamics, parenting difficulties, legal issues and financial constraints, as described by a participant from Wyndham Vale:

She (practitioner) asked me about my relationship and everything, and I’ve discussed … openly like when I’m struggling, what I’m doing and how I’m dealing with it. So, we have discussed not just my depression, my relationship, the difficulties, the difficulties I’m having looking after children, everything.’

In contrast to the whole-person approach, some suggested that being asked questions about adversities in a few instances was uncomfortable and intrusive, viewing these adversities as personal issues. For example, issues like relationship conflicts, partner violence and financial difficulties were perceived as sensitive and personal topics that required vulnerability to discuss. This discomfort arose from the perception that such questions invaded their privacy, making them hesitant to engage openly. This approach felt insensitive because it overlooked the emotional impact of these issues, which underscored more thoughtful and caring questions that acknowledge the emotional weight of these issues and respect individual boundaries.

#### Expectations play a role in talking about adversities

Trusting relationships between caregivers and practitioners created a supportive foundation for ACE inquiries. However, caregivers’ expectations for consultations strongly influenced their willingness to engage in these discussions. Although all caregivers were problem-solving focused, three distinct expectation types emerged: some caregivers sought broad support for their family, some focused exclusively on support for their children and others prioritised assistance with medical issues only.

For caregivers who sought comprehensive support for their children and family, they explained that discussing a wide range of life challenges could facilitate a better understanding of their situation and thus obtain tailored support. One participant from Wyndham Vale noted,

I want issues resolved, so the best way is just to talk and listen and get ideas about my situation.’

Similarly, another from Marrickville said,

I guess ADHD (attention-deficit/hyperactivity disorder) doesn’t happen in a box, so we talk about the other challenges in life that are perhaps impacting the family.’

In contrast, a few participants suggested that the focus of consultation should be on the children instead of the caregivers. As one participant from Wyndham Vale stated,

The primary reason I’m there is for my son, so I’m not talking about me, me, me; it’s all about my boy.’

This view limited the extent and depth of queries about various aspects of the adversities.

A few participants reported that they were visiting the hub to attain services and care related to specific medical problems. Being asked about issues beyond that, such as relationship issues, financial difficulties or mental health challenges, was reported by some participants as uncomfortable. They viewed these issues as ‘embarrassing’ or ‘private’ and suggested that they preferred to manage them independently unless the problem became overwhelming. One participant from Wyndham Vale stated:

I would, for some time, avoid talking about my personal relationships unless it’s overwhelming for me…. But it’s better to talk about anything else than your relationship because I think I would rather do with myself. And if I need help, then I’m going to ask for help.’

#### Barriers to open discussion of adversities

Caregivers were sometimes not reluctant to discuss family or child adversities, but rather lacked opportunities to speak openly. Barriers included hesitancy to discuss life challenges in front of their children, practitioners’ focus on child-specific issues instead of broader family or parental challenges and limited consultation time.

The presence of children when being asked about adversity was a concern for some caregivers and influenced their willingness and extent to share, as described by a participant from Wyndham Vale:

I don’t mind them asking the question, but it depends how much I’d want talk about really in front of the kids.

Some participants suggested that when practitioners provided them with opportunities to share without their children present, it reduced their hesitancy to share. One caregiver from Wyndham Vale explained that they appreciated how the practitioners facilitated her talking about life challenges without the children listening in or being present:

X (practitioner) redirects them [children] to an activity while we have a private conversation. She likes them to do a drawing or like a puzzle while we talk about other things.… She just pulls me aside and like the kids focus, but like it just flows. Like it’s not awkward.

For some consultations, a few caregivers reported little or no scope for bringing up life challenges about their own issues, because the primary focus of practitioners was on their children. One participant from Marrickville stated:

She was more interested in the child but not in us adults as much. So, every time we ask questions to help us … we like to do what we can for our child. We want to be good parents … She would point out a website instead. ‘You can go to this and read it up yourself’. And you’re like, ‘Oh’. You kind of feel dismissed by it.

Another reported that a barrier to discussing adversities ‘in-depth’ was limited consultation time. One participant from Wyndham Vale noted,

Fifteen minutes appointment, so doctor has to push fast … and just listen, write some notes and then get into another one, look after the other patient.

Others echoed this view,

She [practitioner] helps me where she can but doesn’t go too far into depth so that she can put all her time into the paediatric side of it.

While many participants understood that the practitioners were ‘time-poor’, they expressed a need for longer consultation to allow for deeper discussions of life challenges. One participant from Wyndham Vale suggested,

In the ideal world, I think a 30-minute consult would be good, a bit longer.

#### Barriers to accessing services

The magnitude of adversities and daily life activities influenced how participants approached adversity and service navigation. Participants who became exhausted due to the high volume of work and household matters—such as having multiple children, busy work schedules, time constraints and insufficient financial resources—were less likely to connect with the support and care offered. One participant from Wyndham Vale stated,

I was too busy with my children and this work … My MRI came back normal, so I feel like just take painkillers and don’t going for anything.’

Additionally, the limited availability of services and long waitlists jeopardised timely access to the referred services, especially specialised services. One participant from Wyndham Vale shared,

My older child has been on a waiting list for two years to see a certain specialist because they’re too busy … there’s a shortage.’

Another participant from Marrickville highlighted a need for interim support while waiting:

I was struggling because I was not coping with a child who was neurodiverse and not sleeping and etcetera etcetera. It would’ve been good to have some kind of recommendation or referrals to other organisations that perhaps support in that way.

Furthermore, the existing funding model led to high out-of-pocket costs for some caregivers, resulting in limited capacity to use the support plan offered by the practitioners. One participant from Wyndham Vale explained:

One referral was to the X service … but it was going to cost me about $700 for the diagnosis.… I think they weren’t taking any more new clients, and she has referred Z (the child) to the psychologist in Werribee, but they had no availability … So … she referred us to one, the sister counseling, but yeah, they had a hundred and something dollar gap fee, so I didn’t book in with them.

Despite these barriers, some participants reported positive experiences with accessing a range of supports, especially social care services, and material and financial support. Two participants from Wyndham Vale shared:

She [practitioner] put me in touch with a lot of places that could help me with food or petrol vouchers. She put me in touch with the bills that you can get up to $600 paid off your gas, water, and electricity bills. She put me in touch with no-interest places where you can get loans because I had to buy a car…. So, she just put me in touch with lots of useful resources that could help me financially and stuff like that, which was really good.

*She helped with the Centrelink forms to get a carer’s payment for my son … I ended up getting a carer’s allowance and the carer’s payment because of Dr X (practitioner*).

## Discussion

This study explored caregivers’ experiences of being asked about adversities and connected to support and services to address these adversities in CFHs in Australia. Findings indicated that trusting relationships between caregivers and practitioners were fundamental for effective communication about adversities, enabling caregivers to open discussion about adversities and thus access to referred services. However, caregivers’ varying expectations shaped the depth of these discussions—some focused on child-specific issues, while others sought broader family support. Practical barriers, such as the presence of children and limited consultation time, often restricted open discussions. Additionally, caregivers, often overwhelmed by multiple adversities, faced system-related barriers like long waitlists and financial constraints, delaying access to essential support services.

We found that the caregiver-practitioner relationship was central to caregivers’ experiences of care-seeking consultations and navigating services. Preceding studies also identified trusting relationships as a vital element for identifying and responding to service users’ adversities from the perspectives of the practitioners.^42^,^44^ A trusting relationship promoted effective consultations by allowing caregivers to engage in open, meaningful discussions that identified and addressed ACEs, as also found in other studies.^45^,^47^ This trust was fostered by a relational approach, with previous studies highlighting the importance of listening to caregivers, presenting a compassionate manner, paying attention to detail, adopting a nonjudgemental stance and taking a whole-person approach.^4348^,^50^ Specifically, in this study, a whole-person approach encouraged caregivers to share their adversities naturally, especially given that many adversities were perceived as embarrassing and private and were preferred to manage independently by caregivers, as also found in other studies.^51 52^ From practitioners’ perspectives, as Loveday *et al* highlighted, this approach is most effective within an integrated hub model, such as the CFHs, which boosts their confidence by providing a network of services for comprehensive support.^22 42^ We argue that the inputs and supports mobilised at the CFHs through the development and implementation of this model of care helped establish a high level of trust between some caregivers and practitioners.^18 53^

Despite caregivers’ positive experiences of being asked about adversity at the CFH, our findings align with studies conducted in healthcare settings, suggesting a lack of sufficient time to share and respond to adverse events experienced by the caregivers, resulted in minimal engagement or avoidance of asking about adversities or extending discussion points with caregivers.^54 55^ The length of consultations in primary care settings can limit the extent to which practitioners seek to obtain ACE-related information.^33^ A recent review reported that practitioners in US prenatal and primary paediatric care settings typically tend to ask about issues specified in the original ACEs study such as abuse, neglect and household dysfunction.^33^ However, practitioners in Australia’s primary care settings typically have shorter consultation durations which may preclude this. Yet even in a pilot of a screening questionnaire in well-child visits in the United States about ACEs, caregivers felt that the time allocated for their consultations was insufficient to bring up factors situated in and outside the household.^56^ Likewise, the opportunity for being asked about adversities was reported to be less in cases where the caregivers had multiple adversities. Our preceding paper described this as ‘opening Pandora’s box’, implying that the practitioners were less likely to conduct discussions about issues perceived as too sensitive or complex to manage and link to service navigation.^22 42^

Additionally, caregivers in this study reported some barriers to accessing the support they were referred to, mainly due to supply-side factors, such as long waiting times for referral services and high out-of-pocket expenses. Our preceding paper also found that these factors led to fewer practitioners asking about adversity and fewer caregivers following up with services they were referred to.^22^ This is not surprising as other studies also found similar issues.^57^,^59^ Practitioners, as well as the caregivers in this study, reported that limited availability of services within and outside the hubs, long waiting times and high out-of-pocket costs led to fewer practitioners asking about adversity and fewer caregivers following up with services they were referred to.^22^

This finding adds to the discourse on the importance of a holistic approach in supporting families facing adversities, alongside highlighting the significant challenges that impede effective engagement and service delivery. Further investigation should focus on how implications for enhancing care in integrated health and social care services.

### Strengths and limitations

This study interviewed participants who were receiving care for childhood adversity at health and social care hubs. The participants were recruited from a wide range of ages, genders and socioeconomic statuses, which elicited diverse views and experiences. The use of data from multiple sites allowed us to triangulate sources of data, and coders to increase the trustworthiness of the findings. One possible limitation is that some participants might have been uncomfortable sharing their experiences, views, thoughts and adversities because they felt embarrassed about discussing mental health issues and other adversities. However, experienced interviewers tried to minimise this by supporting participants to explore these issues during the interview. The findings of this study are based on data from an urban setting; therefore, the results may not generalise to rural settings. Nevertheless, the information collected from a wide range of participants provides insight into how the caregivers experienced being asked about ACEs and how this related to the support and services required to address ACEs in CFHs in Australia.

## Conclusions

ACEs are major contributors to the burden of disease and continue to be a significant concern for children’s health and well-being. This study explored a novel approach to co-design, test and evaluate a range of supports and services at the CFHs in Victoria and New South Wales to address these adversities. The findings underscore the importance of the caregiver-practitioner relationship in CFHs, particularly the role of trust and rapport in discussions about ACEs. While many caregivers value the opportunities to discuss a range of adversities, barriers such as limited consultation time and differing expectations about practitioners’ roles persist. These challenges often hindered in-depth discussions about adversities, highlighting areas for improvement.

We recommend extending appointment times, clearer communication about the role of the CFHs and their services, and enhanced support for practitioners in navigating sensitive issues with empathy. Addressing these factors would help caregivers feel more comfortable sharing their life challenges. Improving the availability and accessibility of services may further enhance caregivers’ experiences in identifying and addressing adversity.

## supplementary material

10.1136/bmjopen-2024-086710 online supplemental file 1

## Data Availability

Data are available upon reasonable request.
