# Supplementary material for: Caregivers’ experiences of being asked about adverse childhood experiences and receiving support from an integrated health and social care hub: a qualitative study
Source: BMJ Open. 2025 Feb 5;15(2):e086710. doi: 10.1136/bmjopen-2024-086710 (PMC11800215; doi:10.1136/bmjopen-2024-086710)
Supplement: online supplemental file 1 [file bmjopen-15-2-s001.docx]

**Interview guide**

# Section 1: General experience of interacting with the hub

This interview will ask you a few questions about what it is like getting help for life challenges, the easy and hard things about that.

“When we talk about life challenges, it can be anything including not having enough time, feeling tired or stressed, having housing difficulties, not having enough money, mental illness within the family, relationship conflicts, caring for a child with additional needs or challenging child behaviours”.

You don’t have to talk about the life challenges you were experiencing if you are not comfortable. I am just interested in knowing if anyone has asked you or helped you with the life challenges you were experiencing.

1. Tell me about your **family**.
2. Thinking back the past year, **who have you seen** in the IPC health, Wyndham Vale?
3. Tell me about the **experiences of meeting different people** in the IPC health, Wyndham Vale?
4. Thinking back the past year, when you went to the Child and Family Hub at IPC health, has anyone you met **asked** you about life challenges?

- If **yes**: Can you please tell me a little bit about that experience? *(How was that going? Comfortable? Any other challenges being asked?)*
- If **no**: Would you have liked your health care provider *(or use the role/name of the practitioners the participant met)* to ask you about the life challenges you were experiencing?

1. Has anyone you’ve seen suggested you to see anyone **in or outside** the IPC Health in the past 12 months?

- If **yes**: Can you tell me a little bit more about that? *(Type of services? See the person or not? How did that go? What helped? Anything not went well?/ What stopped you linking to the services? - e.g., personal things, family reasons, or other things related to the Hub or services.)*
- If **no**: Would you have liked your health care provider *(or use the role/name of the practitioners the participant met)* to link you with other services that could offer additional support? *(Why - type of services interested in? Why not?)*

1. Thinking back the past year, have you **referred yourself** to any services or organisations, **inside or outside** the Child and Family Hub, IPC Health, to get extra help for your life challenges?

- If **yes**: How did that go for you? *(Type of services? What helped? Anything not went well?)*
- If **not**, why?

1. Thinking back the past year, have you noticed any **changes** in the types of services or activities available at the Child and Family Hub, IPC Health at Wyndham Vale?

- If **yes:** What changes did you notice? *(How did you nitice? Posters/postcard?)*

Did you **use** these services/facilities? *(How did that go for you? What helped? Anything not went well? / Why not use them?)*

- If **nothing**: Is there anything that you would expect to have in the Child and Family Hub at IPC Health? *(Why?)*

1. Thinking back on your experiences in the Child and Family Hub, IPC Health, was there **anything that made you feel like going there for support**? *(This could be the people you worked with, your relationship or interactions with these people, the collaboration between the people working at IPC health, or anything else you can think of.)*

# Section 2: Wellbeing Coordinator Program (WBC)

## Scenario 1: If a client *wasn’t* referred to the WBC

**Explain what the wellbeing coordinator program is:** The wellbeing coordinator program is a new service at IPC Health, Wyndham Vale. The wellbeing coordinator is someone that works with your family to help you make links to other services and communities to help improve your family’s overall wellbeing.

*Note. Wellbeing can be explained as a state of* ***being comfortable, healthy or happy****. It include various aspects such as* ***physical*** *well-being (e.g., being free from illness or discomfort),* ***mental and emotional*** *well-being (e.g., being able to cope with stress; feeling happy and satisfied with life most of the time), and* ***social*** *well-being (e.g., having good relationships within and beyond the family; not feeling isolated).*

- Were you **aware** of this service?
- If yes, how did you become aware of this?
- If not, would you have found it **helpful** to **know about** the wellbeing coordinator program?
- Would you have found it **helpful** to **be referred to** the wellbeing coordinator program? (why, why not)
- Would you consider **referring yourself** to the wellbeing coordinator program? (why, why not)

## Scenario 2: The client *was referred or self-reffered* to the WBC

1. Can you tell me a bit about your experience with the wellbeing coordinator program? *(Who referred you? How did that go for you? What helped? Anything not going well? Comfortable or not?)*
2. Did the wellbeing coordinator’s support **make any changes/differences** in your child/family's wellbeing? *(How? or Why not? Give an example?)*

*Note. Wellbeing can be explained as a state of* ***being comfortable, healthy or happy****. It include various aspects such as* ***physical*** *well-being (e.g., being free from illness or discomfort),* ***mental and emotional*** *well-being (e.g., being able to cope with stress; feeling happy and satisfied with life most of the time), and* ***social*** *well-being (e.g., having fulfilling relationships within and beyond the family; not feeling isolated).*

# Section 3: Legal Support Service

## Scenario 1: If a client *wasn’t* referred to the legal support team

**Explain what legal support service** **is:** The legal support service is a new service at IPC Health, Wyndham Vale. In this service, lawyers are available to help families with housing issues including rent and tenancy, mortgage stress; relationship matters like family violence, parenting arrangements, property disputes, child safety, and protections; car accident and insurance disputes; bills and fines; birth certificates and passports.

- Were you **aware** of this service?
  - If yes, how did you become aware of this team?
  - If not, would you have found it **helpful to know about** the legal support team?
- *[For those who* ***have*** *legal issues]* Would you have found it **helpful to be referred** to the legal support team? (why, why not)
- *[For those who* ***don’t*** *have legal issues]* If you had any of those challenges the lawyers could help with, would you feel comfortable seeing them?

## Scenario 2: The client *was referred* to the legal support team

1. Can you tell me a bit about your experience with the **lawyer**? *(Who referred you? How did that go for you? What helped? Anything not going well? Comfortable or not?)*
2. Did the lawyer’s support **make any changes/differences** in your child/family's wellbeing? *(How? or Why not? Give an example?)*

*Note. Wellbeing can be explained as a state of* ***being comfortable, healthy or happy****. It include various aspects such as* ***physical*** *well-being (e.g., being free from illness or discomfort),* ***mental and emotional*** *well-being (e.g., being able to cope with stress; feeling happy and satisfied with life most of the time), and* ***social*** *well-being (e.g., having fulfilling relationships within and beyond the family; not feeling isolated).*

# Section 4: Ending question

1. In an ideal world, is there **anything that should be included** in the Child and Family Hub, IPC Health, that can identify/address life challenges? *(Thinking about any life challenges, are there any other services would be useful to have at the Child and Family Hub, IPC Health?)*
2. Is there **anything else** that we haven't talked about that you think **would be really useful** **to talk about**?

# Section 5: Collecting demographic information

- - - 1. What is the **MAIN language** your family speaks at your home?
- English
- Other (please specify): _ _ _ _ _ _ _ _ _ _ _ _ _ _ _ _ _ _ _ _
- I prefer not to answer
  - - 1. Where were you **born**?
- Australia
- Other (please specify): _ _ _ _ _ _ _ _ _ _ _ _ _ _ _ _ _ _ _ _
  - - 1. Do you identify as **Aboriginal and/or Torres Strait Islander**?
- Yes
- No
  - - 1. How much **schooling** did you do?
- End of primary school
- Year 10
- Year 12
- Trade or other certificate-level qualification
- Bachelor degree
- Postgraduate qualification
- I prefer not to answer
  - - 1. Are you happy to tell me your **age**? _ _ _ _ _ _ _
      2. Tell me about your family, **who lives at home with you**?

_ _ _ _ _ _ __ _ _ _ _ _ _ _ _ _ _ _ _ _ _ _ _ _ __ _ _ _ _ _ _ _ _ _ _ _ __ _ _ _ _ _ _

# Section 6: Thanks and confirming email address

- Thank you so much for talking with me today. I appreciate you taking the time to share your experiences with me.
- Can I please confirm that I have your correct email address to send the GiftPay voucher? [read back email address]

# Appendix: Participant Information Sheet

1. What is the **MAIN language** your family speaks at your home?

- English
- Other (please specify): _ _ _ _ _ _ _ _ _ _ _ _ _ _ _ _ _ _ _ _
- I prefer not to answer

1. Where were you **born**?

- Australia
- Other (please specify): _ _ _ _ _ _ _ _ _ _ _ _ _ _ _ _ _ _ _ _

1. Do you identify as **Aboriginal and/or Torres Strait Islander**?

- Yes
- No

1. How much **schooling** did you do?

- End of primary school
- Year 10
- Year 12
- Trade or other certificate-level qualification
- Bachelor degree
- Postgraduate qualification
- I prefer not to answer

1. Are you happy to tell me your **age**? _ _ _ _ _ _ _
2. Tell me about your family, **who lives at home with you**?

_ _ _ _ _ _ __ _ _ _ _ _ _ _ _ _ _ _ _ _ _ _ _ _ __ _ _ _ _ _ _ _ _ _ _ _ __ _ _ _ _ _ _
