# Supplementary material for: Caregivers’ experiences of being asked about adverse childhood experiences and receiving support from an integrated health and social care hub: a qualitative study
Source: BMJ Open. 2025 Feb 5;15(2):e086710. doi: 10.1136/bmjopen-2024-086710 (PMC11800215; doi:10.1136/bmjopen-2024-086710)
Supplement: Supplementary file 2 [file bmjopen-2024-086710.reviewer_comments.pdf]

PEER REVIEW HISTORY

BMJ Open publishes all reviews undertaken for accepted manuscripts. Reviewers are asked to complete a checklist review form and are provided with free text boxes to elaborate on their assessment. These free text comments are reproduced below.

ARTICLE DETAILS

Title (Provisional)

Caregivers' experiences of being asked about adverse childhood experiences and receiving support from an integrated health and social care hub: A qualitative study

Authors

Kabir, Ashraful; Loveday, Sarah; Hiscock, Harriet; Bosward, Rebecca; Ebbett , Wilhelmina; Liu, Hueiming; White, Natalie; Chen, Lingling

---

VERSION 1 - REVIEW

**Reviewer**	**1**
**Name**	**Woodman, Jenny**
**Affiliation**	**UCL, Social Research Institute**
**Date**	**23-Jun-2024**
**COI**	**None**

The authors have collected interesting and important data on the experiences of parents living with ACEs at integrated hubs for family health – not always a population that is easy to recruit for interviews. The data is relevant for best practice implementation and guidance for primary care and community health hubs, of which there are increasing amounts not just in Australia but in other countries too, e.g. England (Family Hubs, Women's health hubs). There's lots of potential in this paper but it would benefit from a deeper more complete analysis to explain and connect the themes and generate conclusions relevant to routine questioning about ACEs in health settings. Some detailed notes below.

Abstract

1. Would be helpful to have a definition of ACEs in the abstract – most useful is probably a sentence in methods stating which kinds of ACEs were experienced by the 29 families you spoke to.

Background

2. The authors could explain the relationship of routine questioning about ACEs (the focus of the paper) and "effective communication" in their background section, as they are exchangeable concepts.

The paper would benefit from a logic model or similar showing the programme theory of the hubs – some of this is implied in the background but needs clarity. Having a logic model would allow the reader to see why getting the routine questioning right was so important to support families with ACEs in the hubs and where this fits in the programme theory. It may be that the best place for the logic model is methods. Or it may be that the author see their study as part of the development of the programme and implementation theory and therefore it may go into the results section - detailed programme and implementation theory of these types of complex service models tend to be generated after or alongside implementation and may vary from site to site.

In the background, there is this sentence "One of the critical aspects of this model of care is to identify individuals who have experienced ACEs and 88 address their needs to improve their conditions". But we could do with some further detail – what do you mean by "conditions"? Material conditions e.g. by making sure families have all the welfare payments they are entitled to? Immigration security via legal advice? Legal support with custody battles etc? Supporting parents to improve the home learning environment? Physical health of parents – and what is covered by that? This is important as the activities and mechanisms of change for the intervention (the hub) will be different (maybe overlapping?) across different outcomes. It seems that the aim of the hub may be all these things, given the long list of professionals in the hub. Given the complexity of the hub, the logic model may need to be a simplified one.

Methods

3. The authors need to put in a bit more methods/explanation for the 'baseline survey' which was used to sample participants rather than just referencing another paper. For example, how many respondents were there in the baseline survey and how many reported ACEs, across what domains?

4. The methods would benefit from a table of local areas and interview characteristics across the two sites - e.g. N and average duration of interviews. This could be incorporated into Table 1 and would cut loads of words and make reading easier. Table 1 should include an indication of the range and N of adversities (life challenges) reported by the participants, data should be available from the baseline survey.

Public and patient involvement

5. The authors state that there was no public or patient involvement in this study. What about the pilot interviews with parents reporting ACEs?– I think constitutes public involvement as they had opportunity to shape the language "life challenges" and possibly other content of interviews. It is disappointing to see such little public involvement – I wonder if the authors have done more but have failed to put it here. If not, they should consider involving more parents with adversity now, at the end stage to check and shape interpretation of findings.

Results

6. This is a very relevant and needed study, with sufficient data collected across two sites (allowing comparison) and with purposive sampling to make sure that a range of views and experiences were included. However, the analysis seems a bit superficial and gets stuck in some places at a descriptive level with the themes not building to a coherent whole.

Some examples below of where the analysis could be more developed in terms of depth, moving beyond description and connections between themes.

• In the 'trusting relationships' theme, the main results seem to be that participants appreciate a warm judgemental manner, with the deeper analysis coming at the end with an implied suggestion that a holistic approach was helpful in building trust i.e. asking about family life and infant/child care and nurture as a whole rather than direct questioning about ACEs. The authors could do more to analysis what exact created (did not) a trusting relationship beyond the individual characteristics and manner of the practitioner. For example, in the discussion we find out that the "insensitive" questions mentioned by one participant were attempts at direct questioning on ACEs: "They perceived being questioned directly about issues such as relationship conflicts, partner violence, and financial difficulties as personal and sensitive." but this is not explored and explained in the results. I would like to see more connection between themes in the results– e.g. was the warm, welcoming manner generated by open questions across the child and family system. I would also say that the theme on parental expectations and material circumstance is also connected to building trust and also (probably) to the skill of the practitioner in recognising the starting point and expectations of the parent in front of them and framing their enquiry and needs assessment in light of this. Is the "trusting relationship" the central organising theme here? Or is the material circumstances actually the central organising theme, from which everything else is explained in terms of willingness and capacity to engage with the services.

• Comparison between sites: one of this study's strengths is the dual sites and two hubs (as the authors say in their Strengths and Limitations section). But where is this comparison and "triangulation" woven into the analysis? Were there the same or different views across sites? The interviews were certainly shorter and fewer in one site – does that mean these families felt less positively about the service than families In the other site or where they more likely to have English as a second language or other factor that made recruitment and interviews more tricky?

• Making sense of variation and conflicting views: the authors say that some parents appreciated a whole family approach and others just wanted the 'help' for their child. What does a close look at the data say about why these families felt differently? It has limited utility to say that some families thought x and others y without making sense of it using the data

• Making sense of variation and conflicting views: the authors say that some parents appreciated a whole family approach and others just wanted the 'help' for their child. What

does a close look at the data say about why these families felt differently? It has limited utility to say that some families thought x and others y without making sense of it for the reader using the data. Some of this is done in the discussion rather than as part of the fabric of the analysis e.g. the “lack of confidence” in support services being connected to reluctance to disclose a picture of family life. This type of connection, depth and analysis should be brought into results and substantiated with quotes.

• Alternatives: what did the multiple adversities and difficult material circumstances families say about where else/what else they would want?

• Child protection? There is a complete absence of talking about child welfare in this paper – some ACEs e.g. domestic violence and substance misuse move very quickly into the child protection zone. In other cases, difficult material circumstances and maternal mental health can contribute to difficulties in providing adequate medical, physical or emotional care for the child. How do the families feel about the relationship of practitioners in the hub to child protection. There is a huge body of literature describing the threat experienced by families facing adversity in terms of talking about their family life and potential or actual child protection referrals/activity.

Discussion

7. I would have like the authors to consider what their paper adds or where it challenges other literature as well as where it aligns with other work. There is other literature which could be cited about taking a holistic approach across family life/care in order to support parents facing adversity in caring for their child e.g. this study I was involved in: https://www.ncbi.nlm.nih.gov/pmc/articles/PMC10923434/

8. In terms of strengths and limitations: is this a sample that really has socioeconomic variation? Most of the sample have a university degree.

Conclusions

9. Conclusions can be more aligned with question of “how” to routinely about and support families with ACEs in a hub type model rather than just a statement about “whether” it can be done

---

**Reviewer**	**2**
**Name**	**Barnett, Whitney**
**Affiliation**	**University of Cape Town Department of Paediatrics and Child Health, Paediatrics & Child Health**
**Date**	**04-Sep-2024**
**COI**	**None**

Thank you for this well written paper. I have one minor comment and a few additional suggestions for editing to strengthen the discussion and conclusions.

Minor comment:

- On page 9, “Table 1 summaries” should rather be “Table 1 summarizes”

Other comments:

- The average duration of interviews for each site differed quite a bit (54 vs 23 minutes) and I wonder if you think this may impact the themes which emerged or if that should be discussed in the limitations?

- Similarly, several demographic characteristics were very different between the sites, specifically education, language, and country of birth. I do think this needs to be discussed somewhere. For example, it is not clear to me if the final sample was representative of the target population for CFHs.

- In addition, given these differences, I can understand why table 1 is split between sites but then there isn’t any discussion about why these were split or how themes differed (if they did).

- The conclusion seems to indicate that the CFHs are working well, though it acknowledges some differences of opinion from qualitative interviews, I wonder if more time couldn’t be spent indicating where improvements could be made. The conclusion ends with “Despite supply-side … We argue that this integrated hub model of care approach enables practitioners to build trusting relationships (which are key to helping caregivers make sense of or resolve their adversities) and offer the support and services required within and outside the hubs.” But the conclusion could more directly address how qualitative findings could be translated into improvements in care e.g., longer appointment times, clarity of expectations for the role of the hub (and alignment with the needs of families).

VERSION 1 - AUTHOR RESPONSE

Reviewer: 1

Miss Jenny Woodman, UCL

Comments to the Author:

The authors have collected interesting and important data on the experiences of parents living with ACEs at integrated hubs for family health – not always a population that is easy to recruit for interviews. The data is relevant for best practice implementation and guidance for primary care and community health hubs, of which there are increasing amounts not just in Australia but in other countries too, e.g. England (Family Hubs, Women’s health hubs). There’s lots of potential in this paper but it would benefit from a deeper more complete analysis to explain and connect the themes

and generate conclusions relevant to routine questioning about ACEs in health settings. Some detailed notes below.

Abstract

1. **Comment:** Would be helpful to have a definition of ACEs in the abstract – most useful is probably a sentence in methods stating which kinds of ACEs were experienced by the 29 families you spoke to.

Response*: We have added the following sentence in the abstract section in the revised manuscript to describe the types of ACEs faced by the caregivers [****Row: 25-28; Page: 2****]:*

'A qualitative study design was used. Using a semi-structured interview guide, 29 in-depth interviews were conducted with caregivers of children who were experiencing a range of adversities including maltreatment and household dysfunction, child neglect, parent mental illness, domestic violence, family conflict, community dysfunction, discrimination, poverty or financial hardship'

Background

2. **Comment:** The authors could explain the relationship of routine questioning about ACEs (the focus of the paper) and "effective communication" in their background section, as they are exchangeable concepts.

Response*: Thank you for your insightful comment. We have inserted the following text in the background section of the revised manuscript to clarify the relationship between routine questioning about Adverse Childhood Experiences (ACEs) and effective communication [****Row: 104-114; Page: 5****]:*

'It is recognised that routine inquiries about ACEs are essential for effective communication within integrated care models. By consistently asking about these experiences, service providers convey that caregivers' experiences are valued, fostering trust and rapport. This approach encourages caregivers to actively participate and collaborate in the care process, making effective communication and routine questioning closely linked concepts. Such communication can enhance service users' engagement in the care-seeking process (19, 20), foster collaborative relationships and shared decision-making between service users and providers (21, 22), aid in identifying health issues, develop treatment pathways, and improve adherence to care outcomes (23, 24).'

Comment: The paper would benefit from a logic model or similar showing the programme theory of the hubs – some of this is implied in the background but needs clarity. Having a logic model would

allow the reader to see why getting the routine questioning right was so important to support families with ACEs in the hubs and where this fits in the programme theory. It may be that the best place for the logic model is methods. Or it may be that the author see their study as part of the development of the programme and implementation theory and therefore it may go into the results section - detailed programme and implementation theory of these types of complex service models tend to be generated after or alongside implementation and may vary from site to site.

***Response**: We appreciate the suggestion to include a logic model to clarify the program theory of the Child and Family Hubs (CFHs) Fig 1. We did develop a program logic model for our project and as such, have now included this in the Methods section of the revised paper. This model helped illustrate the significance of effective questioning for supporting families facing ACEs and demonstrate how various components of the CFH work together to address these needs. We have included a figure of the model and added the following texts in the method section [**Row: 176-186; Page: 8**]:*

'The CFH initiative aimed to improve child mental health by identifying and addressing adversities faced by families with children aged 0-8 years. This broader project involved co-designing, implementing, and evaluating CFH models at two sites, focusing on their impact on caregivers' ability to identify adversities and make service referrals. The initiative raised local providers' awareness of ACEs and enhances referral pathways to community hubs and other services. Key activities included training community providers to recognize and respond to ACEs and facilitating referrals within and beyond the CFH. Early detection was intended to increase family participation in CFH, promote collaboration across sectors, and enhance service accessibility. Additionally, the program promoted parent engagement, raised awareness among policymakers about ACE mitigation, and encouraged equitable policies for sustainable scaling. The model also evaluated implementation and outcomes to guide policy changes for sustainability and scalability (18).'

Comment: In the background, there is this sentence "One of the critical aspects of this model of care is to identify individuals who have experienced ACEs and 88 address their needs to improve their conditions". But we could do with some further detail – what do you mean by "conditions"? Material conditions e.g. by making sure families have all the welfare payments they are entitled to? Immigration security via legal advice? Legal support with custody battles etc? Supporting parents to improve the home learning environment? Physical health of parents – and what is covered by that? This is important as the activities and mechanisms of change for the intervention (the hub) will be different (maybe overlapping?) across different outcomes. It seems that the aim of the hub may be all

these things, given the long list of professionals in the hub. Given the complexity of the hub, the logic model may need to be a simplified one.

***Response**: Thank you for raising this insightful comment. We have supplemented the following texts in the background section of the revised manuscript to clarify this point [**Row: 97-100; Page: 5**]:*

'conditions, such as material conditions (e.g., access to housing and welfare payment), immigration-related legal and financial issues, and physical and mental health of the caregiver and child (18)'

Methods

3. **Comment:** The authors need to put in a bit more methods/explanation for the 'baseline survey' which was used to sample participants rather than just referencing another paper. For example, how many respondents were there in the baseline survey and how many reported ACEs, across what domains?

***Response**: We appreciate the point you raised here. We have added the detail that strengthened the methods section and enhanced understanding of how the survey influenced participant selection [**Row: 196-200; Page: 9**]:*

'The baseline survey recruited a total of 349 caregivers (Victoria: n = 234; NSW: n = 115). Across both sites, 88% reported experiencing one or more family adversities across various domains. Over half reported experience of adversity outside the home and 80%. reported at least one form of adversity inside the home (80%).' Further, in Victoria and NSW, almost 40% and 30%, respectively, experienced five or more adversities of any type (22, 42).'

4. **Comment:** The methods would benefit from a table of local areas and interview characteristics across the two sites - e.g. N and average duration of interviews. This could be incorporated into Table 1 and would cut loads of words and make reading easier. Table 1 should include an indication of the range and N of adversities (life challenges) reported by the participants, data should be available from the baseline survey.

*Response: Thank you for this comment. We have revised Table 1, which includes additional details on adversities reported by participants and the average duration of interviews to improve clarity and conciseness [**Row: 273-276; Pages: 12-13**].*

Public and patient involvement

5. **Comment:** The authors state that there was no public or patient involvement in this study. What about the pilot interviews with parents reporting ACEs?– I think constitutes public involvement as they had opportunity to shape the language "life challenges" and possibly other content of interviews. It is disappointing to see such little public involvement – I wonder if the authors have done more but have failed to put it here. If not, they should consider involving more parents with adversity now, at the end stage to check and shape interpretation of findings.

***Response**: Thank you for your valuable feedback regarding public involvement in our study. While we initially stated that there was no formal public or patient involvement, we acknowledge that the pilot interviews with parents did provide an opportunity for participants to influence the language used, such as the term "life challenges." To describe this, we have supplemented the following texts in the revised manuscript under Public and patient involvement section [**Row: 247-254; Pages: 11**] 'The larger study had extensive participation through co-design and lived experience involvement which influenced the design of this study and the CFH. This included CFH consumer involvement to ensure the term 'life challenges' was used throughout our evaluation and not 'ACEs' or 'adversity'.'*

Results

6. This is a very relevant and needed study, with sufficient data collected across two sites (allowing comparison) and with purposive sampling to make sure that a range of views and experiences were included. However, the analysis seems a bit superficial and gets stuck in some places at a descriptive level with the themes not building to a coherent whole.
Some examples below of where the analysis could be more developed in terms of depth, moving beyond description and connections between themes.

• **Comment:** In the 'trusting relationships' theme, the main results seem to be that participants appreciate a warm judgemental manner, with the deeper analysis coming at the end with an implied suggestion that a holistic approach was helpful in building trust i.e. asking about family life and infant/child care and nurture as a whole rather than direct questioning about ACEs. The authors could do more to analysis what exact created (did not) a trusting relationship beyond the individual characteristics and manner of the practitioner. For example, in the discussion we find out that the "insensitive" questions mentioned by one participant were attempts at direct questioning on ACEs: "They perceived being questioned directly about issues such as relationship conflicts, partner violence, and financial difficulties as personal and sensitive." but this is not explored and explained

in the results. I would like to see more connection between themes in the results– e.g. was the warm, welcoming manner generated by open questions across the child and family system. I would also say that the theme on parental expectations and material circumstance is also connected to building trust and also (probably) to the skill of the practitioner in recognising the starting point and expectations of the parent in front of them and framing their enquiry and needs assessment in light of this. Is the "trusting relationship" the central organising theme here? Or is the material circumstances actually the central organising theme, from which everything else is explained in terms of willingness and capacity to engage with the services.

*Response: Thank you for your valuable feedback. We appreciate your recognition of the relevance and depth of our data collection across two sites. In response to your comments about the analysis, we have enhanced our exploration of the "trusting relationships" theme by delving deeper into the factors that create trust beyond practitioner characteristics. We have clarified how a holistic approach and open-ended questions contribute to building trust, as opposed to direct inquiries about ACEs. Additionally, we have drawn clearer connections between themes, particularly the role of parental expectations and material circumstances in fostering trust. We have reflected on your comments by performing a further analysis and reorganise the results section of the revised manuscript [**Row: 293-590; Page: 14-28**]. Thank you for encouraging us to strengthen this aspect of our work.*

• **Comment:** Comparison between sites: one of this study's strengths is the dual sites and two hubs (as the authors say in their Strengths and Limitations section). But where is this comparison and "triangulation" woven into the analysis? Were there the same or different views across sites? The interviews were certainly shorter and fewer in one site – does that mean these families felt less positively about the service than families In the other site or where they more likely to have English as a second language or other factor that made recruitment and interviews more tricky?

Response: Thank you for your valuable comment on the comparison between the two sites. We appreciate your recognition of the dual-site approach as a strength of our study. Although we aimed to present data from both Wyndham Vale and Marrickville, it proved challenging to ensure that their perspectives were equally reflected. The selected quotations were chosen for their ability to best illustrate participants' experiences rather than to provide a direct comparison. We recognize that the shorter interviews and fewer participants in one site may indicate differing experiences or challenges, such as language proficiency or recruitment issues. We agree that these factors may be further investigated in future research.

• **Comment:** Making sense of variation and conflicting views: the authors say that some parents appreciated a whole family approach and others just wanted the 'help' for their child. What does a close look at the data say about why these families felt differently? It has limited utility to say that some families thought x and others y without making sense of it using the data. Some of this is done in the discussion rather than as part of the fabric of the analysis e.g. the "lack of confidence" in support services being connected to reluctance to disclose a picture of family life. This type of connection, depth and analysis should be brought into results and substantiated with quotes.

Response: Thank you for your insightful comment on the varied perspectives of our participants. We appreciate your insight into the importance of understanding the underlying reasons for these differing views. In qualitative research, it is common practice to capture and reflect on a range of perspectives, for gaining a comprehensive understanding of the phenomenon being studied.

While many participants valued the 'whole family approach,' others expressed a preference for more targeted support for their child. Upon taking a deeper look into the data to better explain these variations in viewpoints, we recognize that various factors such as individual family dynamics, prior experiences with services, and specific needs likely influenced their perspectives.

• **Comment:** Alternatives: what did the multiple adversities and difficult material circumstances families say about where else/what else they would want?

Response: Our analysis identified five main themes about caregivers' experiences at both sites, highlighting their complex needs. While many participants appreciated the holistic approach of the hubs, some wanted more specific help for their children. This difference often came from individual situations, like prior experiences and family dynamics, which shaped their expectations and comfort levels during consultations. Some caregivers expressed a need for extra resources to tackle challenges beyond just child-focused support. While they appreciated practitioners, who understood their broader situations, they also wanted practical solutions for immediate concerns.

• **Comment:** Child protection? There is a complete absence of talking about child welfare in this paper – some ACEs e.g. domestic violence and substance misuse move very quickly into the child protection zone. In other cases, difficult material circumstances and maternal mental health can contribute to difficulties in providing adequate medical, physical or emotional care for the child. How do the families feel about the relationship of practitioners in the hub to child protection. There is a huge body of literature describing the threat experienced by families facing adversity in terms of talking about their family life and potential or actual child protection referrals/activity.

Response: Our data did not reflect child protection, likely due to the specific focus and complexity of the topic. We recognize that issues like domestic violence and substance misuse intersect with child protection, and that difficult circumstances can affect caregivers' ability to provide adequate care. While we aimed to cover various aspects of adverse childhood experiences, we agree that not all dimensions can be covered in a single study. Of note, none of our families directly raised the issue of child protection in the interviews.

Discussion

7. **Comment:** I would have like the authors to consider what their paper adds or where it challenges other literature as well as where it aligns with other work. There is other literature which could be cited about taking a holistic approach across family life/care in order to support parents facing adversity in caring for their child e.g. this study I was involved in: https://www.ncbi.nlm.nih.gov/pmc/articles/PMC10923434/

*Response: Thank you for your valuable feedback. We have reviewed the discussion section. We have now incorporated text highlighting the importance of a holistic approach, along with your recommended reference. We have added the following texts to in the discussion section of the revised manuscript [**Row: 655-669; Page: 30**]:*

'This finding adds to the discourse on the importance of a holistic approach in supporting families facing adversities, alongside highlighting the significant challenges that impede effective engagement and service delivery. Further investigation should focus on these dynamics and their implications for enhancing care in integrated health and social care services.'

8. **Comment:** In terms of strengths and limitations: is this a sample that really has socioeconomic variation? Most of the sample have a university degree.

Response: Thank you for this comment. While the majority of our sample did complete university education, 22 to 30% of participants had completed Year 12 (final year of schooling) in Australia only. Thus, whilst many had gone on to compete a university degree, these and others (trade completion) had not which we believe does represent a range of SES.

Conclusions

9. **Comment:** Conclusions can be more aligned with question of "how" to routinely about and support families with ACEs in a hub type model rather than just a statement about "whether" it can be done

*Response: Thank you for your comment. We have received a similar comments and feedback from the second reviewer. We have revised the conclusion section as follows [**Row: 520-536; Page: 24-25**]:*

"ACEs are significant contributors to the burden of disease and remain a substantial concern for the health and wellbeing of children. Our study adopted a novel approach to co-design, test, and evaluate a range of supports and services at Child and Family Hubs (CFHs) in Victoria and New South Wales to address these adversities. Our study highlighted the critical relationship between the caregivers and practitioners within the CFHs, emphasizing the importance of trust and rapport in discussing ACEs and accessing referred services. The findings revealed that while many caregivers valued the opportunities to disclose a range of issues, notable barriers remain including limited consultation time and varying expectations regarding the role of the practitioners. Challenges in accessing referred services included caregivers' limited resources, such as time, social support and finances, along with availability and accessibility of services, especially in terms of medical and mental health areas. These factors often jeopardised comprehensive discussions about adversities and access to needed services, suggesting the need for improvements.

We argue that improvements could include longer appointment times and clearer communication about a hub's role and functions, and increased availability of services are required to enhance the caregivers' experiences. Moreover, facilitating practitioners' ability to navigate sensitive issues with empathy and clarity could help build deeper connections, allowing the caregivers to feel more comfortable to share their challenges. Ultimately, our findings indicate that whilst the hub model of care has strengths, a more nuanced approach aligning with the service delivery with the caregivers' needs is crucial. By realising these improvements, the caregivers can be provided with better support in managing adversities and ensure that their voices are critical in shaping care pathways.'

Reviewer: 2

Mrs. Whitney Barnett, University of Cape Town Department of Paediatrics and Child Health

Comments to the Author:

Thank you for this well written paper. I have one minor comment and a few additional suggestions for editing to strengthen the discussion and conclusions.

Minor comment:

Comment: On page 9, "Table 1 summaries" should rather be "Table 1 summarizes"

*Response: We have replaced the word 'summarizes' with 'summaries' [**Row: 258; Page: 12**]:*

Other comments:

Comment: The average duration of interviews for each site differed quite a bit (54 vs 23 minutes) and I wonder if you think this may impact the themes which emerged or if that should be discussed in the limitations?

*Response: Thank you for your comment. We appreciate your attention to the interview duration differences. While we believe that this variation did not significantly impact the themes that emerged, we have included the following texts to explain this point [**Row: 229-231; Page: 10**]:*

'Although the average duration of interviews varied between sites (54 vs. 23 minutes), we did not observe this to substantially impact the themes that emerged. However, we acknowledge this difference as a minor limitation that may have influenced the depth of insights captured.'

Comment: Similarly, several demographic characteristics were very different between the sites, specifically education, language, and country of birth. I do think this needs to be discussed somewhere. For example, it is not clear to me if the final sample was representative of the target population for CFHs.

Response: Thank you for your insightful comment. We acknowledge the significant demographic differences between the two Child and Family Hubs (CFHs), particularly in education, language, and country of birth. 'We believe these differences did not significantly impact the themes that emerged or our overall analysis.'

Regarding final sampling, we have followed the scientifically accepted procedure described in the 'study participants and sampling strategy' in method section of revised manuscript. The final sample size was considered representative based on the principle of data saturation.

Comment: In addition, given these differences, I can understand why table 1 is split between sites but then there isn't any discussion about why these were split or how themes differed (if they did).

Response: Thank you for your insightful comment. We presented demographic characteristics in a single table as a common and accepted practice in qualitative research. This enables us to provide a clear and concise overview of the participant backgrounds. This format presented the contextual difference and facilitated easy comparison between the two Child and Family Hubs (CFHs). This structured approach supports the rigor of our analysis and provides essential context for interpreting the qualitative data. We have described how these demographic differences impacted the themes emerged or over all analysis in the previous comment.

Comment: - The conclusion seems to indicate that the CFHs are working well, though it acknowledges some differences of opinion from qualitative interviews, I wonder if more time couldn't be spent indicating where improvements could be made. The conclusion ends with "Despite supply-side … We argue that this integrated hub model of care approach enables practitioners to build trusting relationships (which are key to helping caregivers make sense of or resolve their adversities) and offer the support and services required within and outside the hubs." But the conclusion could more directly address how qualitative findings could be translated into improvements in care e.g., longer appointment times, clarity of expectations for the role of the hub (and alignment with the needs of families).

***Response**: We appreciate this insightful comment. We have received the similar kind of feedback and comment form another reviewer. We have revised the conclusion section as follows [**Row: 697-712; Page: 30**]:*

'ACEs are significant contributors to the burden of disease and remain a substantial concern for the health and wellbeing of children. Our study adopted a novel approach to co-design, test, and evaluate a range of supports and services at Child and Family Hubs (CFHs) in Victoria and New South Wales to address these adversities. Our study highlighted the critical relationship between the caregivers and practitioners within the CFHs, emphasizing the importance of trust and report in discussing about ACEs. The findings revealed that while many caregivers valued the opportunities to disclose a range of issues, notable barriers remain including limited consultation time and varying expectations regarding the role of the practitioners. These factors often jeopardised comprehensive discussions about adversities, suggesting the need for improvements.
We argue that longer appointment time and clearer communication hub's role and functions and availability of services required to enhance the caregivers' experiences. Moreover, facilitating practitioner's ability to navigate sensitive issues with empathy and clarity could help build deeper connections, allowing the caregivers to feel more comfortable to share the challenges. Ultimately, our finding indicate that the hub model of care has strengths, a more nuanced approach aligning with the service delivery with the caregivers' needs is crucial. By realising these improvements, the caregivers can be provided better support in managing adversities and ensure that their voices are critical in shaping care pathways.'

Reviewer: 1

If you have selected 'Yes' above, please provide details of any competing interests.: None

Reviewer: 2

If you have selected ‘Yes’ above, please provide details of any competing interests.: None

VERSION 2 - REVIEW

**Reviewer**	**2**
**Name**	**Barnett, Whitney**
**Affiliation**	**University of Cape Town Department of Paediatrics and Child Health, Paediatrics & Child Health**
**Date**	**27-Nov-2024**
**COI**	

Thank you for this article and I appreciate the revisions you have made to improve this. Just a few additional comments below.

1. Page 7: “Over half reported experience of adversity outside the home and 80%. reported at least one form of adversity inside the home (80%).” 80% is repeated.

2. Page 15: “‘I don't mind them asking the question, but it depends how much I'd want talk about really in front of the kids’.” All other quotes are indented and separate from the manuscript text.

3. Page 21, I think report should be rapport? “emphasizing the importance of trust and report in discussing about ACEs.”

4. Page 19. I am struggling a bit with context, in the discussion you state “Our findings suggest that caregivers faced some barriers to openly discussing their adversities in depth and accessing the support they were referred, mainly due to supply-side factors, such as insufficient time for clinical consultations, long waiting times for referral services, and high out-of-pocket expenses.” But just before that also cite the importance of CFHs that “providing a network of services for comprehensive support” I understand that needs will exist beyond the scope of services offered by CFHs but am wondering if you have a sense of whether patients were seeing multiple staff at the CFH to receive more comprehensive services vs being referred out and how this impacted their experience? In fact it looks like the qualitative interview has sub-sections about what services participants were referred to, so this could be quantified. I think that would add some value and context in terms of understanding service utilization (by clinic) but also whether this impacted themes.

5. Page 21, row 451 to 451, the first sentence of that paragraph needs a bit of editing for clarity/grammar. Actually a few places in that last paragraph, rather than “the challenges” perhaps “their challenges” and instead of “Ultimately, our finding” rather “findings” and

then I am not sure what is meant by that first clause … our finding indicate that the hub has strengths, it is not clear to me that this is the case and if so what those strengths are (from the current research presented)? Sorry, the last two sentences don’t make sense to me either.

VERSION 2 - AUTHOR RESPONSE

Reviewer: 2
Mrs. Whitney Barnett, University of Cape Town Department of Paediatrics and Child Health
Comments to the Author:
Thank you for this article and I appreciate the revisions you have made to improve this. Just a few additional comments below.

Comment 1: Page 7: “Over half reported experience of adversity outside the home and 80%. reported at least one form of adversity inside the home (80%).” 80% is repeated.

Response: Thank you for your feedback. We have removed the repeated texts in the revised manuscript as follows [Row: 160-161; Page: 7]:

‘Over half reported experiencing adversity outside the home, and 80% reported at least one form of adversity inside the home.’

Comment 2: Page 15: “‘I don't mind them asking the question, but it depends how much I'd want talk about really in front of the kids’.” All other quotes are indented and separate from the manuscript text.

Response: Thank you for your feedback. We have adjusted the formatting of the quote to be indented and separated, ensuring consistency throughout the manuscript.

Comment 3: Page 21, I think report should be rapport? “emphasizing the importance of trust and report in discussing about ACEs.”

Thank you for pointing out this typo. We have corrected it to "rapport" in the revised manuscript.

Comment 4: Page 19. I am struggling a bit with context, in the discussion you state “Our findings suggest that caregivers faced some barriers to openly discussing their adversities in depth and accessing the support they were referred, mainly due to supply-side factors, such as insufficient time for clinical consultations, long waiting times for referral services, and high out-of-pocket expenses.” But just before that also cite the importance of CFHs that “providing a network of services for comprehensive support” I understand that needs will exist beyond the scope of services offered by CFHs but am wondering if you have a sense of whether patients were seeing multiple staff at the CFH to receive more comprehensive services vs being referred out and how this impacted their experience? In fact it looks like the qualitative interview has sub-sections about what services participants were referred to, so this could be quantified. I think that would add some value and context in terms of understanding service utilization (by clinic) but also whether this impacted themes.

Response: Thank you for your thoughtful comment. We appreciate your suggestion to explore how patients utilised multiple staff at the CFHs versus being referred out for services.

We have revised the last two paragraphs of the discussion to ensure the logic of the two arguments is consistent: Although some caregivers had positive experiences of being asked about adversities and linked to services within the CFHs, which provided a network of comprehensive support, some caregivers

experienced barriers to discussing their adversities and/or connecting to referred services, such as supply-side factors. Please find the revision in Rows 401-422, pages 19-20.

We understand the value of quantifying service utilisation and explore how it may impact caregivers' experiences. However, given the qualitative nature of the study, the service utilisation questions in the interview schedule were used to elicit caregivers' memory of care seeking experience rather than accurately quantifying their service utilisation. As a result, we would cautiously use these data as their accuracy may impact the interpretation of how service utilisation may impact their experiences.

Additionally, we believe that the current results already provide sufficient context regarding caregivers' experiences within the CFHs and their referral processes.
We hope that the current framing provides adequate clarity regarding how service provision and referrals impacted caregivers' experiences.

Comment 5: Page 21, row 451 to 451, the first sentence of that paragraph needs a bit of editing for clarity/grammar. Actually a few places in that last paragraph, rather than "the challenges" perhaps "their challenges" and instead of "Ultimately, our finding" rather "findings" and then I am not sure what is meant by that first clause … our finding indicate that the hub has strengths, it is not clear to me that this is the case and if so what those strengths are (from the current research presented)? Sorry, the last two sentences don't make sense to me either.

Response: Thank you for your comment. We have revised the relevant text in the conclusion section for clarity in the revised manuscript, as follows [Row: 442-456; Page: 21]:

'ACEs are major contributors to the burden of disease and continue to be a significant concern for children's health and wellbeing. This study explored a novel approach to co-design, test, and evaluate a range of supports and services at the CFHs in Victoria and New South Wales to address these adversities. The findings underscore the importance of the caregiver-practitioner relationship in CFHs, particularly the role of trust and rapport in discussions about ACEs. While many caregivers valued the opportunities to discuss a range of adversities, barriers such as limited consultation time and differing expectations about practitioners' roles persist. These challenges often hindered in-depth discussions about adversities, highlighting areas for improvement.

We recommend extending appointment times, clearer communication about the role of the CFHs and their services, and enhanced support for practitioners in navigating sensitive issues with empathy. Addressing these factors would help caregivers feel more comfortable sharing their life challenges. Improving the availability and accessibility of services may further enhance caregivers' experiences in identifying and addressing adversity..'

Reviewer: 2
If you have selected 'Yes' above, please provide details of any competing interests.: Not applicable
Date Sent: n/a

VERSION 3 - REVIEW

**Reviewer**	**2**
**Name**	**Barnett, Whitney**
**Affiliation**	**University of Cape Town Department of Paediatrics and Child Health, Paediatrics & Child Health**
**Date**	**13-Dec-2024**

COI

Thank you for your responsive edits and congrats on this paper!
